# Macrophage iron dyshomeostasis promotes aging‐related renal fibrosis

**DOI:** 10.1111/acel.14275

**Published:** 2024-07-17

**Authors:** Lingzhi Wu, Hongchun Lin, Shaomin Li, Yuebo Huang, Yuxiang Sun, Shuangshuang Shu, Ting Luo, Tiantian Liang, Weiyan Lai, Jialing Rao, Zhaoyong Hu, Hui Peng

**Affiliations:** ^1^ Nephrology Division, Department of Medicine, the Third Affiliated Hospital Sun Yat‐sen University Guangzhou China; ^2^ Nephrology Division, Department of Medicine Baylor College of Medicine Houston TX USA; ^3^ NHC Key Laboratory of Clinical Nephrology (Sun Yat‐sen University) and Guangdong Provincial Key Laboratory of Nephrology Guangzhou China

**Keywords:** aging‐related renal fibrosis, ferroptosis, iron dyshomeostasis, macrophage, pcbp1, rutin, single‐cell RNA sequencing data, stat1

## Abstract

Renal aging, marked by the accumulation of senescent cells and chronic low‐grade inflammation, leads to renal interstitial fibrosis and impaired function. In this study, we investigate the role of macrophages, a key regulator of inflammation, in renal aging by analyzing kidney single‐cell RNA sequencing data of C57BL/6J mice from 8 weeks to 24 months. Our findings elucidate the dynamic changes in the proportion of kidney cell types during renal aging and reveal that increased macrophage infiltration contributes to chronic low‐grade inflammation, with these macrophages exhibiting senescence and activation of ferroptosis signaling. CellChat analysis indicates enhanced communications between macrophages and tubular cells during aging. Suppressing ferroptosis alleviates macrophage‐mediated tubular partial epithelial‐mesenchymal transition in vitro, thereby mitigating the expression of fibrosis‐related genes. Using SCENIC analysis, we infer Stat1 as a key age‐related transcription factor promoting iron dyshomeostasis and ferroptosis in macrophages by regulating the expression of Pcbp1, an iron chaperone protein that inhibits ferroptosis. Furthermore, through virtual screening and molecular docking from a library of anti‐aging compounds, we construct a docking model targeting Pcbp1, which indicates that the natural small molecule compound Rutin can suppress macrophage senescence and ferroptosis by preserving Pcbp1. In summary, our study underscores the crucial role of macrophage iron dyshomeostasis and ferroptosis in renal aging. Our results also suggest Pcbp1 as an intervention target in aging‐related renal fibrosis and highlight Rutin as a potential therapeutic agent in mitigating age‐related renal chronic low‐grade inflammation and fibrosis.

## Graphical abstract

During renal aging, the accumulation of senescent macrophages promotes tubular partial epithelial‐mesenchymal transition through the secretion of the senescence‐associated secretory phenotype, ultimately contributing to age‐related renal fibrosis. We propose that Pcbp1 is a key factor for iron homeostasis, and its downregulation promotes renal macrophage senescence by inducing iron dyshomeostasis. While the transcription factor Stat1 contributes to Pcbp1 downregulation, Rutin can preserve the level of Pcbp1 under senescent conditions. This suggests that Rutin is a promising agent for modulating the function of Pcbp1 during renal aging.

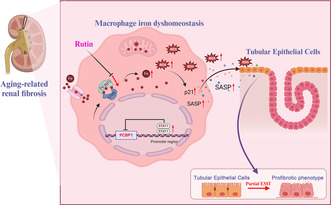

**Abbreviations**
4HNE: 4‐Hydroxynonenal
BLM: bleomycin
DEGs: differentially expressed genes
EMT: epithelial‐mesenchymal transition
Fer‐1: Ferrostatin‐1
GO: Gene Ontology
Gpx4: glutathione peroxidase 4
GSH: glutathione
GSVA: Gene Set Variation Analysis
H_2_O_2_
: hydrogen peroxide
KEGG: Kyoto Encyclopedia of Genes and Genomes
LPO: lipid peroxidation
SASP: senescence‐associated secretory phenotype
SCENIC: Single‐Cell Regulatory Network Inference and Clustering
ScRNA‐seq: single‐cell RNA sequencing
TFRC: transferrin receptor
TFs: transcription factors
UMAP: Uniform Manifold Approximation and Projection

## 1 INTRODUCTION

Aging is characterized by the gradual depletion of functional reserves and a significant decline in the capacity to maintain homeostasis in response to external or internal stresses, leading to an elevated risk of disease and mortality (Fang et al., 2020; Schmitt & Melk, 2017). As the population ages, the imperative tasks of delaying aging and enhancing the quality of life for the elderly have gained prominence.

Under physiological conditions, the kidneys are highly metabolic organs capable of withstanding substantial oxidative stress. However, they are notably susceptible to the aging process. Indeed, the kidneys are among the organs that undergo the most dramatic changes during normal aging (Fang et al., 2020; Schmitt & Melk, 2017; Zhou et al., 2021). An aging kidney often exhibits diminished filtration function, leading to an elevated prevalence of chronic kidney disease (Fang et al., 2022; Luo et al., 2018; Schmitt & Melk, 2017). Given the substantial burden of kidney disease in the elderly, understanding the impact of aging on the kidneys is crucial for developing effective interventions and improving health outcomes.

While multiple homeostatic mechanisms independently contribute to the aging process, age‐related chronic inflammation has emerged as a prevalent factor (Gulen et al., 2023). Senescent cells exhibit the senescence‐associated secretory phenotype (SASP), secreting factors that promote chronic inflammation and induce senescence in normal cells. Simultaneously, this chronic inflammation accelerates immune cell aging, leading to weakened immune responses that are ineffective in clearing senescent cells and inflammatory factors. This creates a detrimental cycle of inflammation and aging (Li et al., 2023). Within the kidneys, aging results from the accumulation of senescent cells and chronic low‐grade inflammation. Such chronic inflammation impairs intrinsic tissue regeneration and exacerbates kidney damage, increasing the susceptibility to aging‐related kidney diseases and diminishing the capacity for kidney regeneration (Sato & Yanagita, 2019).

Therefore, targeting inflammation may represent a potential strategy against renal aging.

A major characteristic of chronic inflammation involves persistent activation of the innate immune system, leading to dysregulation in the secretion of proinflammatory and anti‐inflammatory factors (Teissier et al., 2022). This imbalance is believed to contribute to the aging of solid organs, with monocytes and macrophages being central players in promoting low‐grade chronic inflammation (Yao et al., 2023). With aging, macrophages experience a reduction in phagocytic capacity, diminishing their ability to clear inflammation (Oishi & Manabe, 2016). Concurrently, aging bone marrow‐derived macrophages produce higher levels of proinflammatory cytokines, increasing susceptibility to pathogens (Li et al., 2023; Oishi & Manabe, 2016). Therefore, despite similar levels of inflammatory stimuli, the capacity of older individuals to resolve inflammation is significantly reduced, leading to persistent chronic inflammation.

Aging macrophages retain a degree of plasticity, making them susceptible to changes in autophagy, nutrient perception, and mitochondrial dysfunction related to aging. Recent studies suggest that interventions targeting macrophages could extend human lifespan and reduce susceptibility to aging‐related diseases. Strategies such as caloric restriction and the use of metabolically targeted drugs, including metformin, resveratrol, and rapamycin, have been shown to impact longevity by influencing the macrophage population (Fabbiano et al., 2016; Minhas et al., 2021; van Beek et al., 2019; Vasamsetti et al., 2015). Therefore, targeting macrophage functionality presents a viable approach for improving the inflammatory state associated with aging.

Here, we performed single‐cell RNA sequencing analyses to investigate the dynamic changes in the proportion and function of renal cell types during aging, particularly focusing on the role of chronic low‐grade inflammation. Our findings reveal that macrophages, as key players in this inflammation, are the most abundant and proinflammatory immune cells in aging kidneys. We further demonstrated that these macrophages not only exhibit senescence but also activate ferroptosis signaling, contributing to the perpetuation of chronic low‐grade inflammation and promoting tubular partial epithelial‐mesenchymal transition. In addition, we identified critical transcription factors and genes that regulate this process of macrophage ferroptosis in aging. Finally, our study proposes a potential therapeutic agent aimed at mitigating aging‐related renal fibrosis, possibly by addressing the underlying chronic low‐grade inflammation.

## 2 MATERIALS AND METHODS

### 2.1 Single‐cell transcriptomic data

We employed single‐cell RNA sequencing (scRNA‐seq) data to explore the dynamic changes in the composition and function of renal cell types throughout the aging process in mice. This spanned various developmental stages: 8 weeks, 12, 18, and 24 months. Our analysis utilized single‐cell transcriptomic data from the Cell Landscape database (http://bis.zju.edu.cn/cellatlas/; Wang et al., 2023). We validated our findings with single‐cell transcriptomic data from the Tabula Muris Senis dataset (Tabula Muris Consortium, 2018). We downloaded the original gene‐barcode matrices, aggregated them, and preprocessed them using Seurat v3.0.2 within the R package, we filtered out low‐quality nuclei possessing more than 5000 features or fewer than 500. Furthermore, we excluded nuclei expressing genes in fewer than three nuclei. Subsequently, the filtered matrix underwent normalization and scaling through the application of the “LogNormalize” method and the ScaleData function in Seurat.

### 2.2 ScRNA‐seq data bioinformatics analysis

The Uniform Manifold Approximation and Projection (UMAP) algorithm was applied to achieve cell visualization. To analyze differential gene expression, the Wilcoxon rank‐sum test was employed. The numbers of differentially expressed genes (DEGs) overlapping between the groups (12 months vs. 8 weeks, 18 months vs. 8 weeks, 24 months vs. 8 weeks) were visualized using Venn diagrams. Gene Ontology (GO) enrichment analysis and Kyoto Encyclopedia of Genes and Genomes (KEGG) pathway analysis of the DEGs were conducted using the clusterProfiler package (v3.12.0), with statistical significance set at a *p*‐value <0.05. To assess gene set enrichment in kidney macrophages, we used the “GSVA” R package performing Gene Set Variation Analysis (GSVA) to calculate enrichment scores. CellChat was employed for intercellular communication analysis. Additionally, Single‐Cell Regulatory Network Inference and Clustering (SCENIC) was utilized to infer the transcriptomic factors and genes directly controlled by them in kidney macrophages.

### 2.3 Mice

C57BL/6J male mice were housed in standard environmental conditions, with a regular light/dark cycle, free access to water, and a chow diet. Mice were euthanized at 8 weeks and 24 months of age, and renal tissues were collected for analysis. All animal experiments followed the animal research reporting of in vivo experiments (ARRIVE) guidelines and received approval from the Ethics Committee of South China Agricultural University.

### 2.4 Cell culture and drug‐induced senescent cell models induction

The mouse macrophage cell line RAW264.7 and the rat kidney tubular cell line NRK‐52E were obtained from ATCC. Cells were cultured in DMEM supplemented with 10% FBS and 1% penicillin–streptomycin and maintained in a 37°C incubator with 5% CO_2_. Drug‐induced senescent cell models were established as previously described (Bai et al., 2023; Gulen et al., 2023; Wang et al., 2021). RAW264.7 cells were treated with 300 μmol/L hydrogen peroxide (H_2_O_2_) or 5 μg/mL bleomycin (BLM). The medium was changed every 72 h for 7 days. In a 12‐well transwell system, RAW264.7 cells were added to the upper chamber and treated with H_2_O_2_ or BLM with or without the addition of Ferrostatin‐1 (Fer‐1). After washing away the rest reagents, NRK‐52E cells were added to the lower chamber. After 3 days of co‐culture, RAW264.7 cells were withdrawn and NRK‐52E cells were collected.

### 2.5 Histology examination and immunofluorescence

Fresh renal tissues underwent fixation in 4% paraformaldehyde for at least 24 h at room temperature, then were paraffin‐embedded and sectioned at 4‐μm slices for Masson, Sirius Red, and H&E staining, adhering to the manufacturer's instructions. Images were captured using a Nikon E80 microscope. The collagen‐stained area was calculated as a percentage of the total area. For immunofluorescence, slides were blocked with serum at room temperature for 1 h then incubated with specific primary antibodies overnight at 4°C. These antibodies included anti‐F4/80 (1:100, Santa Cruz Biotechnology, 1:500, Servicebio), anti‐p21 (1:500, Abcam), anti‐IL‐1β (1:4000, Servicebio), anti‐4HNE (1:100, R&D Systems), and anti‐TFRC (1:100, Abcam). The slides were incubated with fluorescently labeled secondary antibodies on the following day and stained nuclei with DAPI.

Images were analyzed using ImageJ software version 1.53.

### 2.6 Lipid peroxidation (LPO) and glutathione (GSH) measurement

Intracellular levels of LPO and GSH were measured using an LPO assay kit (A106‐1) and a reduced glutathione (GSH) assay kit (A006‐2‐1), respectively, following the instructions provided with the products.

### 2.7 Cell ferrous ion (Fe^2+^) level analysis

Following supernatant removal, cells underwent three PBS washes. Subsequently, they were trypsinized and centrifuged at 188 *g* for 5 min. According to the manufacturer's instructions, cells were treated with a FerroOrange working solution (#F374, Dojindo) and incubated for 30 min at 37°C. Fluorescence intensity was then measured using a flow cytometer.

### 2.8 Virtual screening and molecular docking

Virtual screening, a computational method of compound screening based on a small molecule database, was employed. Using the Standard Molecular Property feature in the Maestro11.9 platform, we predicted the physicochemical properties of all compounds derived from the anti‐aging compound entity library (2297 compounds, https://www.selleck.cn/). After filtering and screening, we obtained 2154 compounds. Additionally, the Pcbp1 target protein crystal structure was downloaded from the Protein Data Bank (https://www.rcsb.org/) and processed on the Maestro11.9 platform. Virtual filtering processing and optimization were performed using the Glide module in the Schrodinger Maestro software. Following docking, the top compounds were selected based on the energy score. Twenty compounds were finally chosen through an evaluation of the binding energy score and pivotal residues of the active site.

### 2.9 Immunoblotting

Proteins were extracted from cultured cells and quantified using a BCA protein assay kit (Beyotime, P0010). Subsequently, SDS‐PAGE (Roche, 3010040001) was employed to separate the proteins, and they were transferred onto polyvinylidene fluoride membranes. Blocking of the membranes took place with 5% bovine serum albumin at room temperature for 1 h, followed by an overnight incubation with the corresponding primary antibodies: anti‐p21 (1:1000, Abcam), anti‐Gpx4 (1:1000, Abcam), anti‐gamma H2A.X (phospho S139) (1:1000, Abcam), anti‐Pcbp1 (1:1000, Abclonal), anti‐Fibronectin (Abcam, 1:3000), anti‐Collagen I (Cell Signaling Technology, 1:1000) and anti‐β‐actin (1:2000, Cell Signaling Technology). Corresponding species‐specific secondary antibodies were incubated for 1 h on the following day. Finally, the membranes were visualized using an ECL blot detection reagent.

### 2.10 Real‐time quantitative PCR

Total RNA was extracted using TRIzol reagent (Invitrogen, 15596018). cDNA synthesis was carried out with the PrimeScript™ RT reagent Kit with gDNA Eraser (Perfect Real Time; TaKaRa, RR047A). qPCR was conducted on a Roche LightCycler480 using Taq Pro Universal SYBR qPCR Master Mix (Vazyme, Q712‐02). The primer sequences are listed in Table S1.

### 2.11 Gene silencing or overexpression

The siRNA of Pcbp1 and their matched scramble control were purchased from Gene Pharma (Guangzhou, China). The sense sequence for Pcbp1 siRNA is “5′‐GCUGACUGGGCCUACCAAUTT‐3′.” The antisense sequence is “5′‐AUUGGUAGGCCCAGUCAGCTT‐3′.” The Pcbp1 overexpression was achieved by transfection of pCDH‐CMV‐Pcbp1 expressing plasmid (Umine bio, China) with Lipofectamine 3000 (Thermo Fisher Scientific, L3000015) according to the manufacturer's protocol.

### 2.12 Chromatin immunoprecipitation (ChIP) assay

ChIP assays were performed using the Enzymatic ChIP kit (Cell Signaling Technology). RAW264.7 macrophages were incubated with or without BLM. According to the manufacturer's protocol, chromatin was immunoprecipitated with an anti‐Stat1 antibody or a negative control IgG antibody at 4°C overnight. DNA samples were purified using magnetic beads and amplified using the following primers: F, 5′‐GTCTTGCTCTGTTGGGGAAAAG‐3′ and R, 5′‐TGTGCCACAGCAGATTCCATT‐3′. The PCR products were added to a 1% agarose gel with GelRed nucleic acid gel stain, and images were captured using the Tanon gel documentation system.

### 2.13 Statistical analysis

Statistical analyses were conducted utilizing GraphPad Prism 8.0 and R software version 4.1.2. Image analysis employed ImageJ software version 1.53. Experiments were repeated a minimum of three times. Data, along with error bars, are presented as mean ± SEM. Significance was determined using Student's *t*‐test and one‐way ANOVA, with significance thresholds set at *p < 0.05, **p < 0.01, ***p < 0.001.

## 3 RESULTS

### 3.1 Macrophage infiltration contributing to kidney chronic low‐grade inflammation during renal aging

**FIGURE 1:**
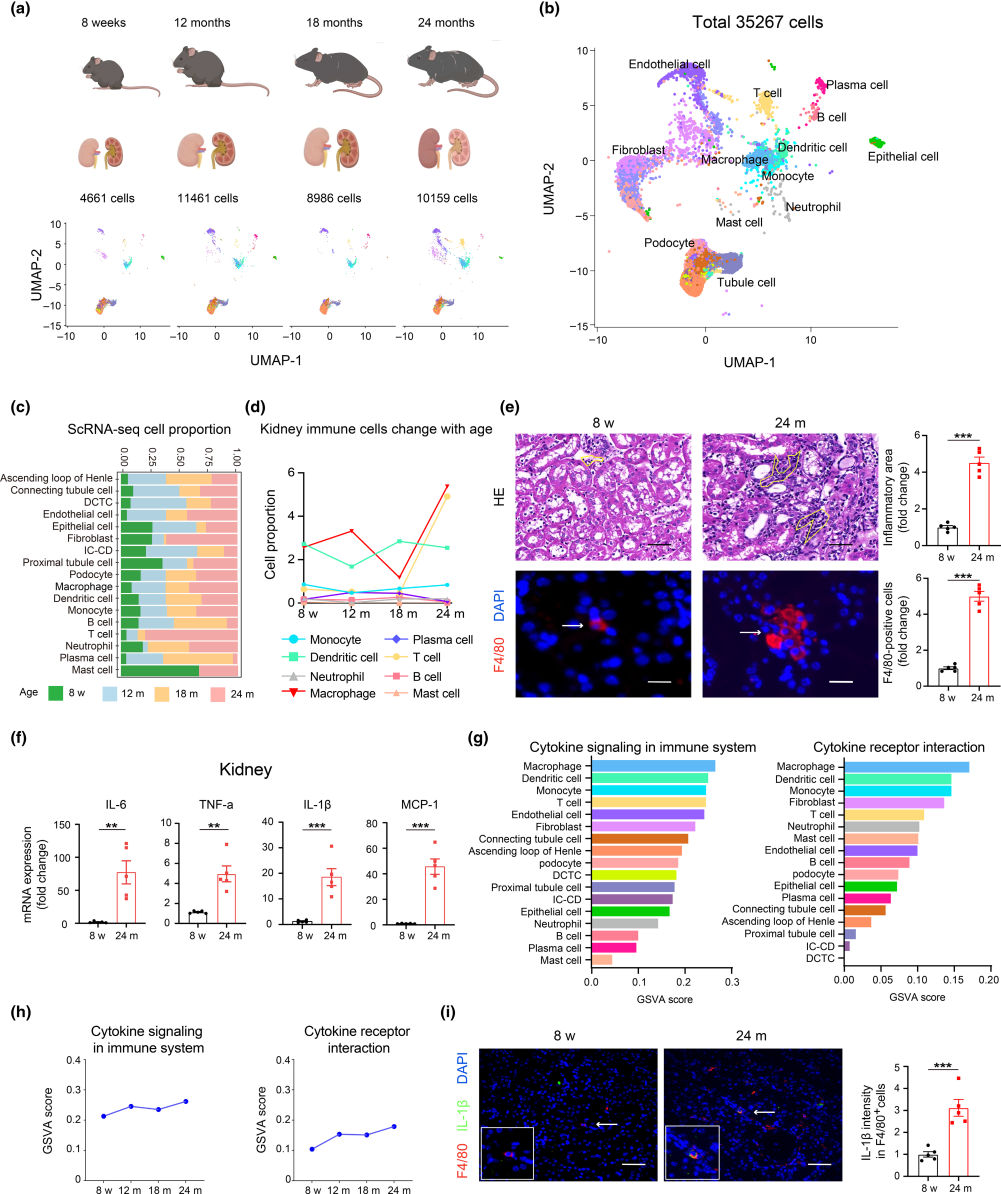
Single‐cell RNA sequencing data revealed increased macrophage inflammation during renal aging. (a, b) UMAP visualization of single cells from kidneys across all stages of mice, colored by cluster identity. Our analysis utilized single‐cell transcriptomic data from the Cell Landscape database. (c) The proportion of various cell types during renal aging. (d) The proportion of major immune cell types changed with renal aging. (e) HE staining in kidney tissues from five young and five aged mice (upper). Representative images and quantification of the inflammatory focus area (yellow line) were shown. Scale bars, 20 μm. Representative immunofluorescence images and quantification analysis of macrophages marker F4/80 (arrow) in kidney tissues (Lower). Scale bars, 20 μm. (f) RT‐qPCR analysis of IL‐6, TNF‐a, IL‐1β and MCP‐1 mRNAs in kidney tissues from five young and five aged mice. (g) GSVA score of the cytokine signaling in immune system and cytokine receptor interaction were calculated for all types of renal cells. (h) GSVA score of the cytokine signaling in immune system and cytokine receptor interaction were calculated for macrophages across the age stage. (i) Representative immunofluorescence images and quantification analysis of co‐staining of IL‐1β (green) and F4/80 (red) (arrow) in kidney tissues from five young and five aged mice. Scale bars, 25 μm. DCTC, distal convoluted tubule cell; IC‐CD, intercalated cell of collecting duct. Data were represented as the mean ± SEM. *p < 0.05, **p < 0.01, and ***p < 0.001.

To delineate the dynamic changes in the proportion and function of various cell types during renal aging, we analyzed scRNA‐seq data from C57BL/6J mice kidneys. This data, sourced from the published online “Cell Landscape Resource” (http://bis.zju.edu.cn/cellatlas/; Wang et al., 2023), spanned four‐time points from the 8 weeks stage to 24 months (Figure 1a). Cell types were annotated based on the expression of canonical cell type‐specific markers. In the overall mouse kidney cell landscape, 17 distinct cell types were identified, including epithelial cells, fibroblasts, endothelial cells, and immune cells (Figure 1b). These cell types showed varying proportions in the kidney across different developmental stages and ages, with a notable increase in immune cell infiltration (Figure 1c). Among these, macrophages were identified as the predominant immune cell type in aging kidneys (Figure 1d). To validate these observations, kidneys from five young (8 weeks old) and five aged (24 months old) C57BL/6J mice were analyzed. Histological analyses confirmed increased immune cell infiltration in aging kidneys (Figure 1e, upper). Elevated macrophage accumulation was further evidenced by immunostaining using the F4/80 marker (Figure 1e, lower). Additionally, SASP‐related factors, including IL‐6, TNF‐α, IL‐1β, and MCP‐1, were upregulated in aged kidneys (Figure 1f), indicating that renal aging is characterized by chronic inflammation. Focusing on macrophages, their role in renal chronic inflammation was further investigated. Gene Set Variation Analysis (GSVA) revealed that macrophages exhibited an increased inflammatory response with age, reaching the highest degree compared to other cell populations in the kidneys of 24‐month‐old mice (Figure 1g,h; Figure S1a,b). Immunostaining showed colocalization of IL‐1β with F4/80, with a higher number of IL‐1β‐positive macrophages in aged mice than in the young group (Figure 1i). These bioinformatics findings, coupled with experimental evidence, demonstrate that chronic low‐grade inflammation characterizes aging kidneys, significantly contributed to by increased macrophage infiltration.

### 3.2 Macrophages exhibit senescence and undergo ferroptosis in renal aging

**FIGURE 2:**
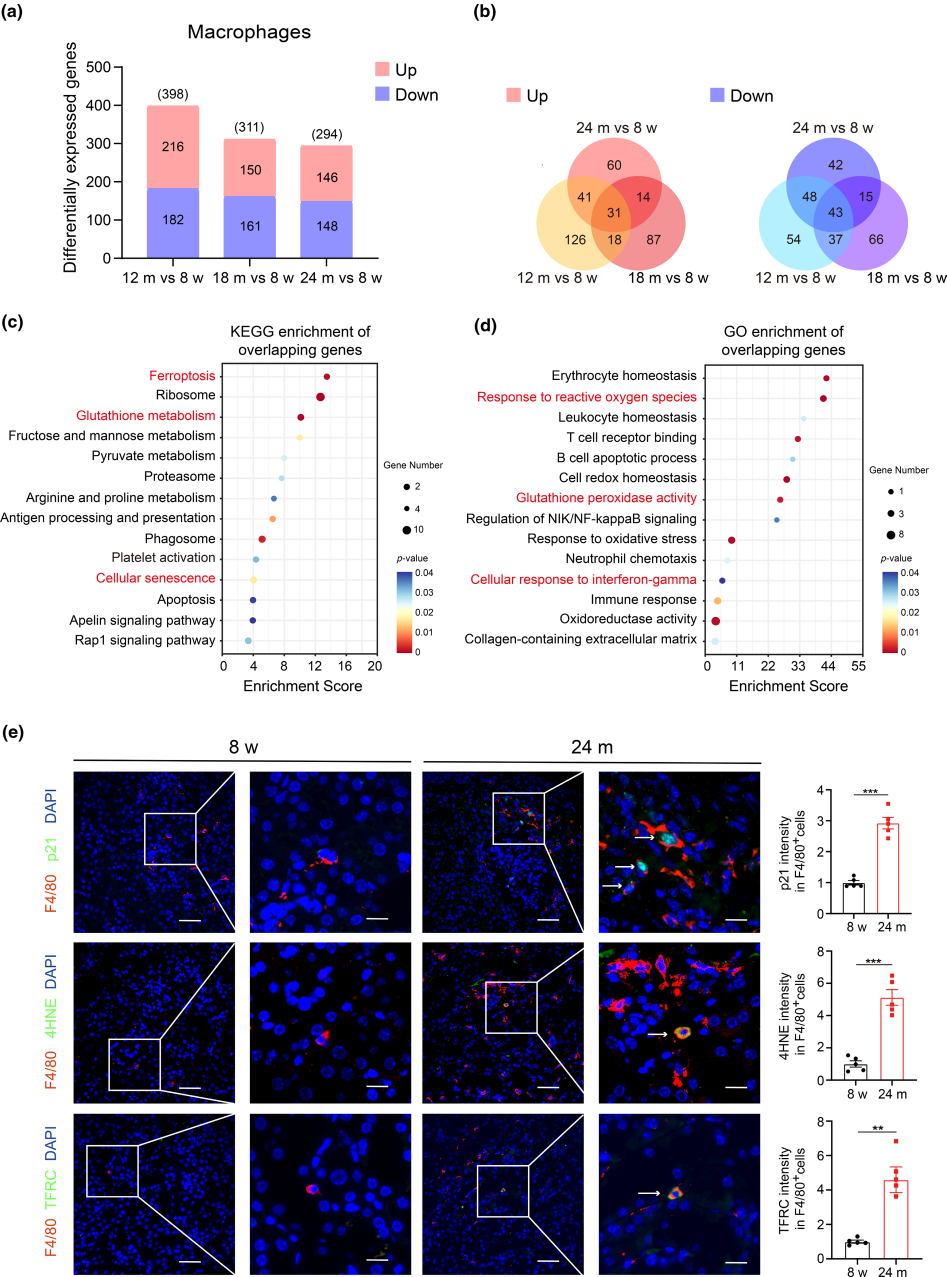
Single‐cell RNA sequencing data revealed macrophages exhibited senescence and underwent ferroptosis in renal aging. (a) DEGs of macrophages in the middle‐aged group (12 months) and aged group (18, and 24 months) compared to the young group (8 weeks). (b) Venn diagram showing the overlapped DEGs of macrophages (left: Upregulation; right: Downregulation). KEGG pathway (c) and GO pathway (d) analysis of the overlapping DEGs of macrophages. (e) Representative immunofluorescence images and quantification analysis of co‐staining of p21 (green) and F4/80 (red) (upper) (arrow), co‐staining of 4HNE (green) and F4/80 (red) (middle) (arrow), co‐staining of TFRC (green) and F4/80 (red) (lower) (arrow) in kidney tissues from five young and five aged mice. Scale bars, 50, 25 μm. DEGs, differentially expressed genes; KEGG, Kyoto Encyclopedia of Genes and Genomes; GO, Gene Ontology; 4HNE, 4‐ Hydroxynonenal; TFRC, Transferrin Receptor. Data were represented as the mean ± SEM. *p < 0.05, **p < 0.01, and ***p < 0.001.

Recognizing the significant role of inflammation in aging, we sought to gain a deeper understanding of the biological changes in macrophages during renal aging. Initially, we selected macrophage clusters and identified differentially expressed genes (DEGs) in aged groups (12, 18, and 24 months) compared to the young group (8 weeks), revealing 398 (12 months), 311 (18 months), and 294 (24 months) DEGs, respectively (Figure 2a). Despite varying expression across different age stages, some DEGs were consistently observed. Specifically, we identified 31 overlapping DEGs that were consistently upregulated and 43 overlapping DEGs that were downregulated during aging (Figure 2b; Figure S2). KEGG analysis revealed that the overlapping DEGs in macrophages were closely associated with ferroptosis (Pcbp1, Hmox1, Sat1), glutathione metabolism, and cellular senescence (Figure 2c). GO analysis indicated enrichment in pathways related to the response to reactive oxygen species, glutathione peroxidase activity, and cellular response to interferon‐gamma (Figure 2d). To validate the reliability of our results, we analyzed another single‐cell sequencing dataset related to kidney aging (Tabula Muris Senis, GSE109774). The KEGG enrichment results indicated that macrophages in 24‐month‐old kidneys were also involved in pathways related to inflammation, ferroptosis, cellular senescence, and glutathione metabolism (Figure S3). Immunofluorescent staining at the protein level validated these findings, showing colocalization of the senescence marker p21 with the macrophage marker F4/80 in aged kidneys, consistent with macrophage senescence observed at the single‐cell transcription level (Figure 2e, upper). Additionally, 4‐Hydroxynonenal (4HNE), a biomarker of lipid peroxidation, was significantly elevated in aged kidney macrophages but barely detected in young kidneys (Figure 2e, middle). The transferrin receptor (TFRC), critical for iron uptake, also colocalized with F4/80 (Figure 2e, lower). In summary, our findings indicate that macrophages undergo senescence and ferroptosis during renal aging.

### 3.3 Enhanced communications between macrophages and tubular cells associated with aging‐related renal fibrosis

**FIGURE 3:**
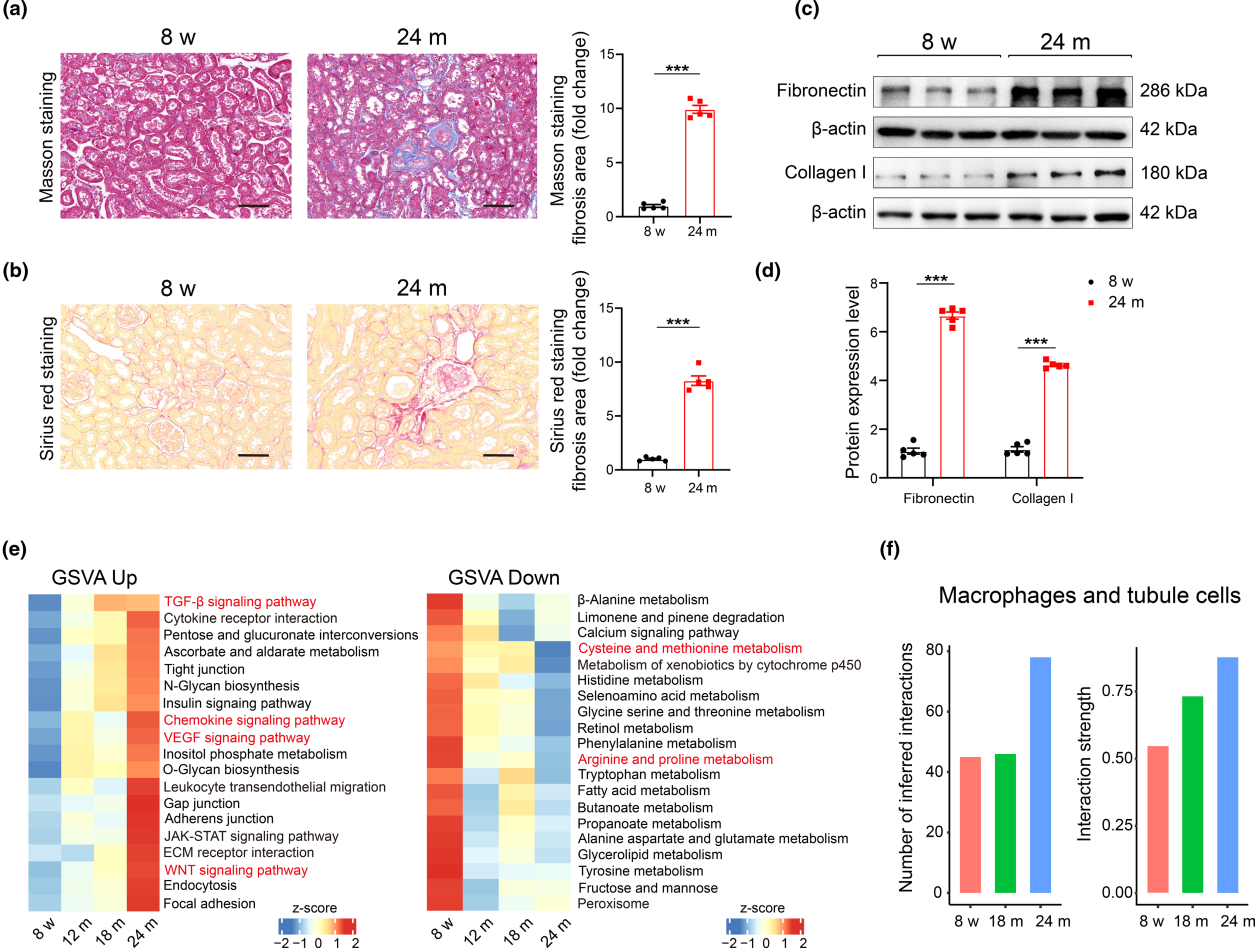
Enhanced communications between macrophages and tubular cells potentially contributed to aging‐related renal fibrosis. (a, b) Representative images and quantitative data for the positive area of fibrosis of Masson's trichrome and Sirius red staining in kidney tissues from five young and five aged mice. Scale bars, 20 μm. Representative western blot (c) and quantification (d) of fibronectin and collagen I protein expression in kidney tissue from five young and five aged mice. (e) Heatmap showing enrichment score of the significantly enriched KEGG gene sets across age stages (left: Up; right: Down). (f) The number of interactions between macrophages and tubule cells by CellChat analysis. Data were represented as the mean ± SEM. *p < 0.05, **p < 0.01, and ***p < 0.001.

Unresolved inflammation is recognized as a central issue in fibrotic diseases, with macrophages playing a pivotal role in the pathophysiological processes of various types of tissue fibrosis, including renal fibrosis, which is a significant manifestation of renal aging (Fu et al., 2022; Jiang et al., 2022; Wynn & Barron, 2010). The aged kidney exhibited prominent interstitial fibrosis, with an observed increase in fibrosis in the tubulointerstitial area (Figure 3a,b). Additionally, the expression of extracellular matrix proteins, such as fibronectin and collagen I, was elevated in aged kidneys (Figure 3c,d). Gene Set Variation Analysis (GSVA) revealed that KEGG gene sets related to fibrotic signalings, such as the TGF‐β, VEGF, and WNT pathways increased with age in macrophages. Concurrently, inflammatory pathways, such as the chemokine signaling pathway and leukocyte transendothelial migration, also demonstrated an increase in response to aging (Figure 3e, left). In contrast, metabolism‐related pathways, including cysteine and methionine metabolism and arginine and proline metabolism, decreased with aging (Figure 3e, right). These results suggest that macrophages maintain a proinflammatory and pro‐fibrotic state during renal aging. Recent studies have shown that iron accumulation drives fibrosis, senescence, and the SASP in the kidney (Maus et al., 2023). Activated macrophages that secrete substantial amounts of TGF‐β can promote fibrosis in tissues, including adipose tissue fibrosis (Yu et al., 2024). Additionally, partial epithelial‐mesenchymal transition (EMT) of renal tubular cells has been suggested as another mechanism underlying kidney fibrosis (Grande et al., 2015). To further elucidate the role of macrophage ferroptosis signaling in aging‐related renal fibrosis, we investigated changes in interactions between macrophages and tubular cells during kidney aging using CellChat analysis. This analysis suggested an increase in interactions between macrophages and tubule cells with aging (Figure 3f). Collectively, these findings indicate significant changes within macrophages, potentially contributing to the pro‐fibrotic microenvironment and aging‐related renal fibrosis.

### 3.4 Suppression of ferroptosis signaling inhibits macrophage‐mediated partial epithelial‐mesenchymal transition in vitro

**FIGURE 4:**
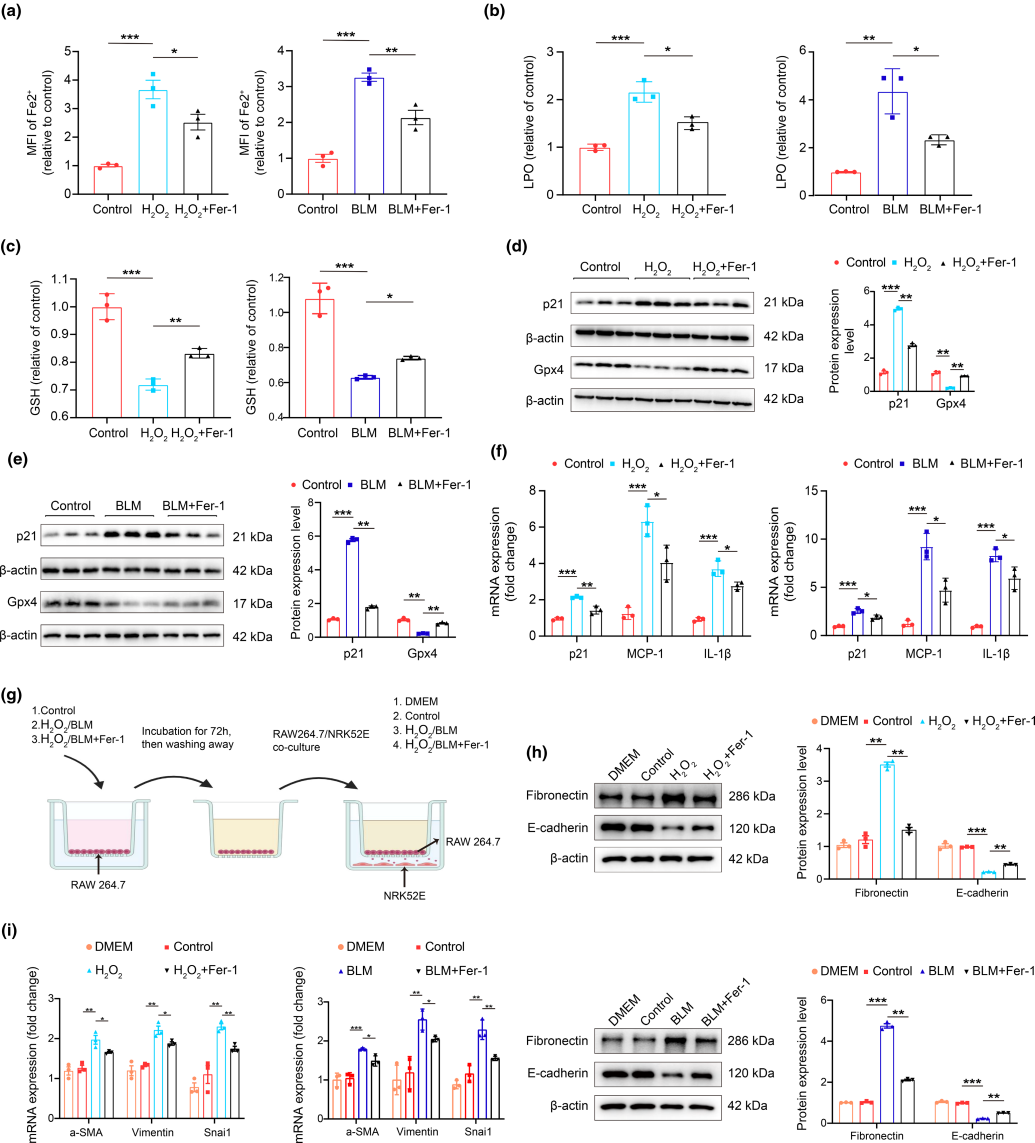
Inhibiting ferroptosis signaling by Ferrostatin‐1 alleviated macrophage‐mediated tubular epithelial‐mesenchymal transition in vitro. Cell ferrous ion (Fe^2+^) level analysis (a), lipid peroxidation measurement (b), and glutathione measurement (c) in drug‐induced senescent macrophage models in vitro with or without Fer‐1. (d, e) Representative western blot and quantification of p21 and Gpx4 protein expression in drug‐induced senescent macrophage models in vitro with or without Fer‐1. (f) RT‐qPCR analysis of p21, MCP‐1, IL‐1β mRNAs in drug‐induced senescent macrophage models in vitro with or without Fer‐1. (g) Schematic diagram of co‐culture of RAW264.7 cells and NRK‐52E kidney tubular cell. (h) Representative western blot and quantification of fibronectin and e‐cadherin protein expression in co‐culture models in vitro (upper and lower). (i) RT‐qPCR analysis of α‐SMA, Vimentin, and Snai1 mRNA in drug‐induced senescent macrophage models in vitro with or without Fer‐1. Data were represented as the mean ± SEM. *p < 0.05, **p < 0.01, and ***p < 0.001.

Given the increasing recognition of the pro‐fibrotic microenvironment and the heightened intercellular communications between macrophages and tubule cells, we investigated whether suppressing ferroptosis signaling could inhibit macrophage‐mediated partial epithelial‐mesenchymal transition (EMT). To this end, we established in vitro models of drug‐induced senescent cells and conducted cell co‐culture experiments (Bai et al., 2023; Gulen et al., 2023; Wang et al., 2021). Hydrogen peroxide (H_2_O_2_) treatment simulates oxidative stress‐induced senescence, providing a model to study oxidative stress responses in cells (Bai et al., 2023). Bleomycin (BLM) is a chemotherapy agent that also serves as an inducer of cellular senescence due to its ability to cause DNA damage, disrupting DNA metabolism (Gulen et al., 2023). Specifically, RAW264.7 macrophages were treated with 300 μmol/L H_2_O_2_ or 5 μg/mL BLM to induce senescence, resulting in increased expression of p21, γH2A.X (Figure S4a,b). These results confirmed the establishment of our drug‐induced macrophage senescence models. We then explored the effects of Ferrostatin‐1 (Fer‐1) on macrophage senescence. Fer‐1 is a potent inhibitor of ferroptosis, known for its effectiveness in blocking lipid peroxidation, a process catalyzed by free iron and critical to the execution of ferroptosis. Gpx4 (Glutathione Peroxidase 4) is a crucial enzyme in the ferroptosis pathway. It prevents ferroptosis by reducing lipid hydroperoxides to lipid alcohols, thereby inhibiting the formation of Fe^2+^‐dependent reactive oxygen species (ROS; Bayır et al., 2020). Treatment with Fer‐1 reduced cell ferrous ion levels and lipid peroxidation (LPO) levels, while increasing glutathione (GSH) levels (Figure 4a–c). Additionally, Fer‐1 suppressed the expression of p21, MCP‐1, and IL‐1β, while stimulating Gpx4 expression (Figure 4d–f). These results suggest that suppressing ferroptosis signaling could alleviate macrophage senescence and reduce inflammatory factor levels. Finally, we investigated the impact of ferroptosis inhibition in macrophages on the phenotypic changes of tubular cells. RAW264.7 macrophages were co‐cultured with NRK‐52E tubular cells, as outlined in Figure 4g. Senescence was induced in RAW264.7 macrophages using H_2_O_2_ or BLM, with or without the addition of Fer‐1. After thoroughly washing, these cells were incubated with strainer‐containing NRK‐52E cells for 72 h. As shown in Figure 4h, fibronectin protein levels increased in the presence of senescent macrophages, but this response was suppressed by Fer‐1 treatment. Conversely, decreased E‐cadherin levels, indicative of EMT in tubule cells, were observed in the presence of senescent macrophages, but this response was reversed after Fer‐1 treatment. In addition, EMT markers such as α‐SMA, Vimentin, and Snai1 increased at the mRNA level in the presence of senescent macrophages but decreased following Fer‐1 treatment (Figure 4i). In summary, our findings suggest that inhibiting ferroptosis signaling in macrophages can alleviate tubular partial epithelial‐mesenchymal transition, implicating a potential pathway to reduce or prevent fibrosis in aging kidneys.

### 3.5 Identification of key genes in ferroptosis during macrophage immunosenescence and renal aging

**FIGURE 5:**
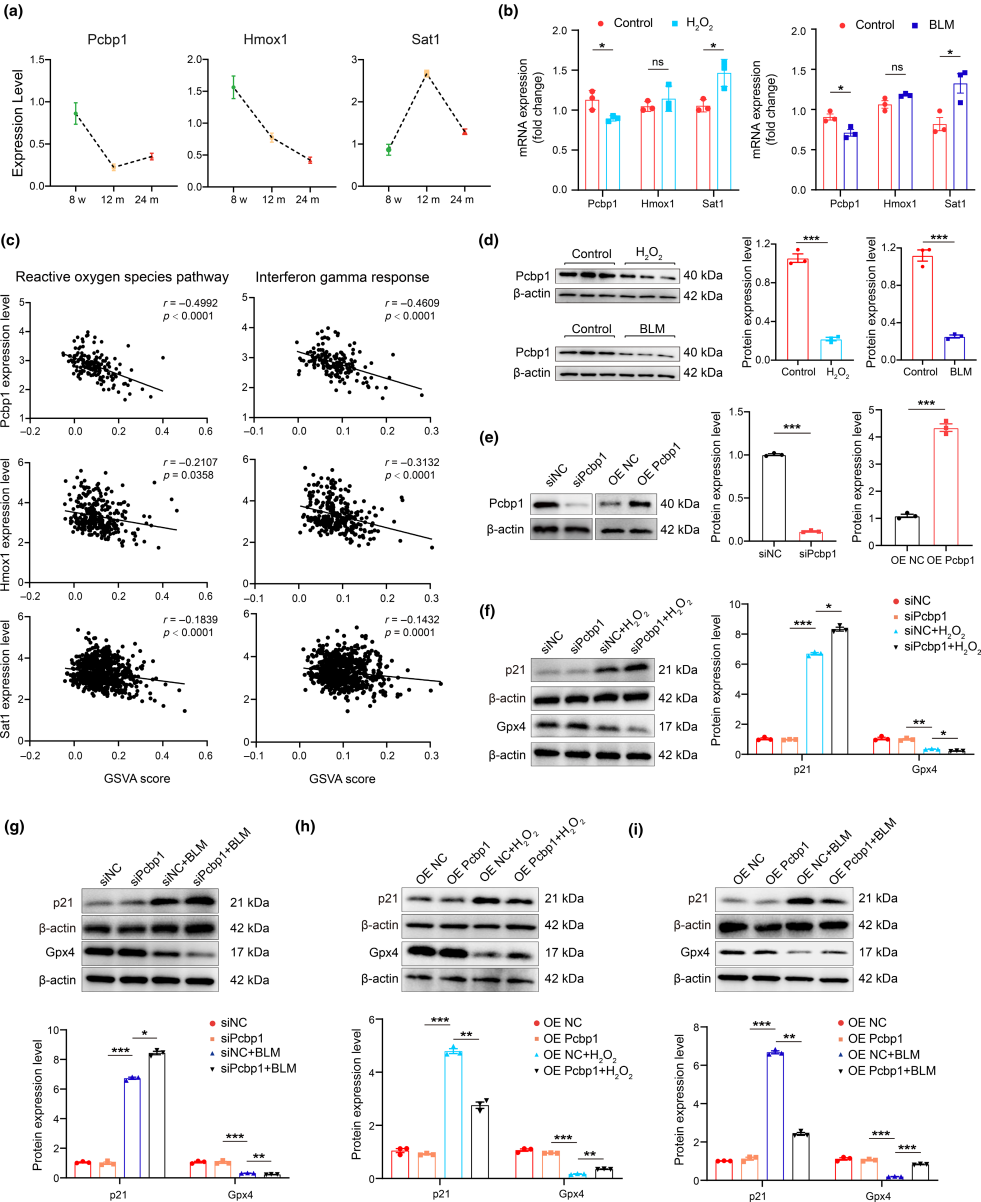
Pcbp1 was the core gene in macrophage senescence and ferroptosis. (a) Gene expression levels of overlapped ferroptosis‐related genes from single‐cell RNA sequencing data. (b) RT‐qPCR analysis of Pcbp1, Hmox1, and Sat1 mRNAs in drug‐induced senescent macrophage models in vitro. (c) Significant correlations between the Pcbp1, Hmox1, and Sat1 expression level and the GSVA score of reactive oxygen species pathway and interferon‐gamma response pathway in single‐cell RNA sequencing data. (d) Representative western blot and quantification of protein expression of Pcbp1 in drug‐induced senescent macrophage models in vitro. (e) The transfection efficiency of Pcbp1 overexpressed plasmid or pcbp1 siRNA. (f, g) Transfected Pcbp1 siRNA with H_2_O_2_ and BLM stimulation. Representative western blot and quantification analysis of protein expression of p21 and Gpx4 in macrophages were shown. (h, i) Transfected Pcbp1 overexpression plasmid with H_2_O_2_ and BLM stimulation. Representative western blot and quantification analysis of protein expression of p21 and Gpx4 in macrophages were shown.

After establishing the crucial role of ferroptosis signaling in macrophage immunosenescence and age‐related renal fibrosis, we aimed to identify key genes involved in the ferroptosis process. ScRNA‐seq analysis revealed progressive changes in the expression of ferroptosis‐related genes, including Pcbp1, Hmox1, and Sat1, in kidney macrophages with aging. Specifically, Pcbp1 and Hmox1 expression decreased with age, while Sat1 expression increased (Figure 5a). In vitro studies using senescent macrophage models confirmed the mRNA expression patterns of Pcbp1 and Sat1, with no significant alterations observed in the expression of Hmox1 (Figure 5b). Further functional correlation analysis showed that Pcbp1 expression was more closely associated with the reactive oxygen species pathway, interferon‐gamma response pathway, and leukocyte transendothelial migration pathway than either Hmox1 or Sat1 (Figure 5c; Figure S5). Additionally, Pcbp1 protein expression significantly decreased with the progression of senescence (Figure 5d). Since Pcbp1 functions as an iron chaperone, capable of carrying and distributing free iron in the form of glutathione‐linked ferrous iron (GSH‐Fe II) and facilitating various biological redox reactions, we inferred that Pcbp1 is a crucial anti‐ferroptosis gene in regulating macrophage ferroptosis signaling.

Next, we explored the impact of Pcbp1 on senescence in macrophages by transfecting cells with either Pcbp1 siRNA or a Pcbp1 overexpression plasmid, followed by stimulation with H_2_O_2_ and BLM. The efficiency of knockdown or overexpression was demonstrated in Figure 5e. Our results revealed that Pcbp1 knockdown resulted in decreased Gpx4 and increased p21 protein levels (Figure 5f,g). Conversely, Pcbp1 overexpression led to reduced p21 protein expression and elevated Gpx4 protein expression in the presence of H_2_O_2_ or BLM (Figure 5h,i). These findings suggest that Pcbp1 functions as a crucial anti‐ferroptosis gene, potentially offering new therapeutic targets for mitigating age‐related macrophage immunosenescence.

### 3.6 Stat1 as a key regulator modulating Pcbp1 in macrophages during renal aging

**FIGURE 6:**
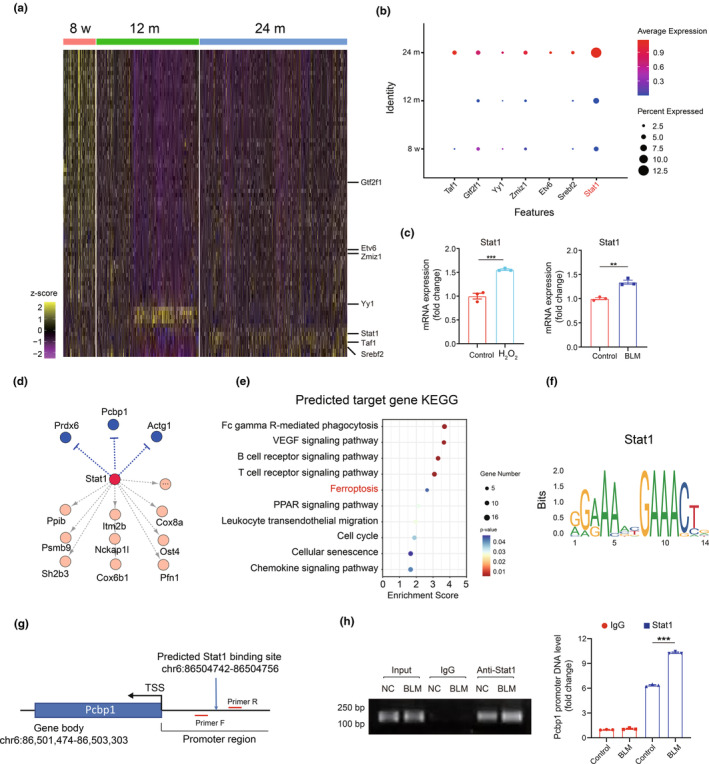
Stat1 was the key transcription factor in macrophage senescence and ferroptosis by SCENIC analysis. (a) Heatmap showing the age‐related transcription factors in macrophages during aging by SCENIC analysis. (b) The expression of Taf1, Gtf2f1, Yy1, Zmiz1, Etv6, Srebf2, and Stat1 in the single‐cell RNA sequencing data. (c) RT‐qPCR analysis of Stat1 mRNAs in the drug‐induced senescent macrophage models in vitro. (d) Unsupervised regulator analysis showed that Pcbp1 is within the regulon of Stat1. Yellow means up, blue means down. (e) KEGG enrichment analysis of predicted target gene of Stat1. (f) Binding motifs of Stat1 from the JASPAR database. (g) A schematic illustration depicts the Stat1 motif within the promoter region of the Pcbp1 locus. (h) ChIP assay analysis of Stat1 binding to Pcbp1 in macrophages treated with or without BLM. Data were represented as the mean ± SEM. *p < 0.05, **p < 0.01, and ***p < 0.001.

To understand the gene regulatory networks in aging kidneys and identify potential transcription factors (TFs) modulating the differentially expressed genes (DEGs) in response to aging in the kidney, we conducted a Single‐Cell Regulatory Network Inference and Clustering (SCENIC) analysis. This allowed us to infer TFs and their directly controlled genes, known as “regulons” in the macrophage cluster. The SCENIC analysis yielded 52 TFs and 73 regulons (Table S2). We selected several TFs to highlight in Figure 6a, including Taf1, Gtf2f1, Yy1, Zmiz1, Etv6, Srebf2, and Stat1. In parallel, we surveyed the mRNA expression of these TFs and found that Stat1 was significantly upregulated in 24‐month‐old mice (Figure 6b). Stat1 expression was increased in the macrophage senescence models induced by H_2_O_2_ and BLM, consistent with single‐cell sequencing data (Figure 6c; Figure S6). Unsupervised regulator analysis indicated that Pcbp1 is within the regulon of Stat1, suggesting it is directly regulated by Stat1 in macrophages (Figure 6d). Notably, most genes in the Stat1 regulon are upregulated, but several genes such as Pcbp1 are downregulated during aging. This indicates that Stat1 can act as either a transcription activator or repressor during aging (Cao et al., 2023). Pathway enrichment analysis of Stat1 target DEGs highlighted its roles in Fc gamma R‐mediated phagocytosis, the VEGF signaling pathway, ferroptosis, PPAR signaling pathway, leukocyte transendothelial migration, cell cycle, and cellular senescence (Figure 6e). These results suggest that Stat1 acts as an upstream hub governing macrophage phenotype in aged kidneys.

We further analyzed the information on Stat1 from the JASPAR database and visualized the consensus sequence “GAAA … GAAACT”, likely the binding site for Stat1 (Figure 6f). We inferred a Stat1 binding site within the promoter region of the Pcbp1 locus (Figure 6g). A ChIP assay confirmed that Stat1 can regulate the expression of Pcbp1 by binding to its promoter: under normal conditions, enriched Pcbp1 promoter DNA fragments were observed in the immunoprecipitation using an anti‐Stat1 antibody. However, BLM treatment which induces ferroptosis in macrophages, significantly intensified this response, indicating that senescence stimulates Stat1 binding to the Pcbp1 promoter, resulting in downregulation of Pcbp1 expression (Figure 6h). In summary, our results suggest the involvement of Stat1 in regulating Pcbp1 expression during macrophage ferroptosis.

### 3.7 Molecular docking and virtual screening identifying Rutin as a potential agent against renal aging

**FIGURE 7:**
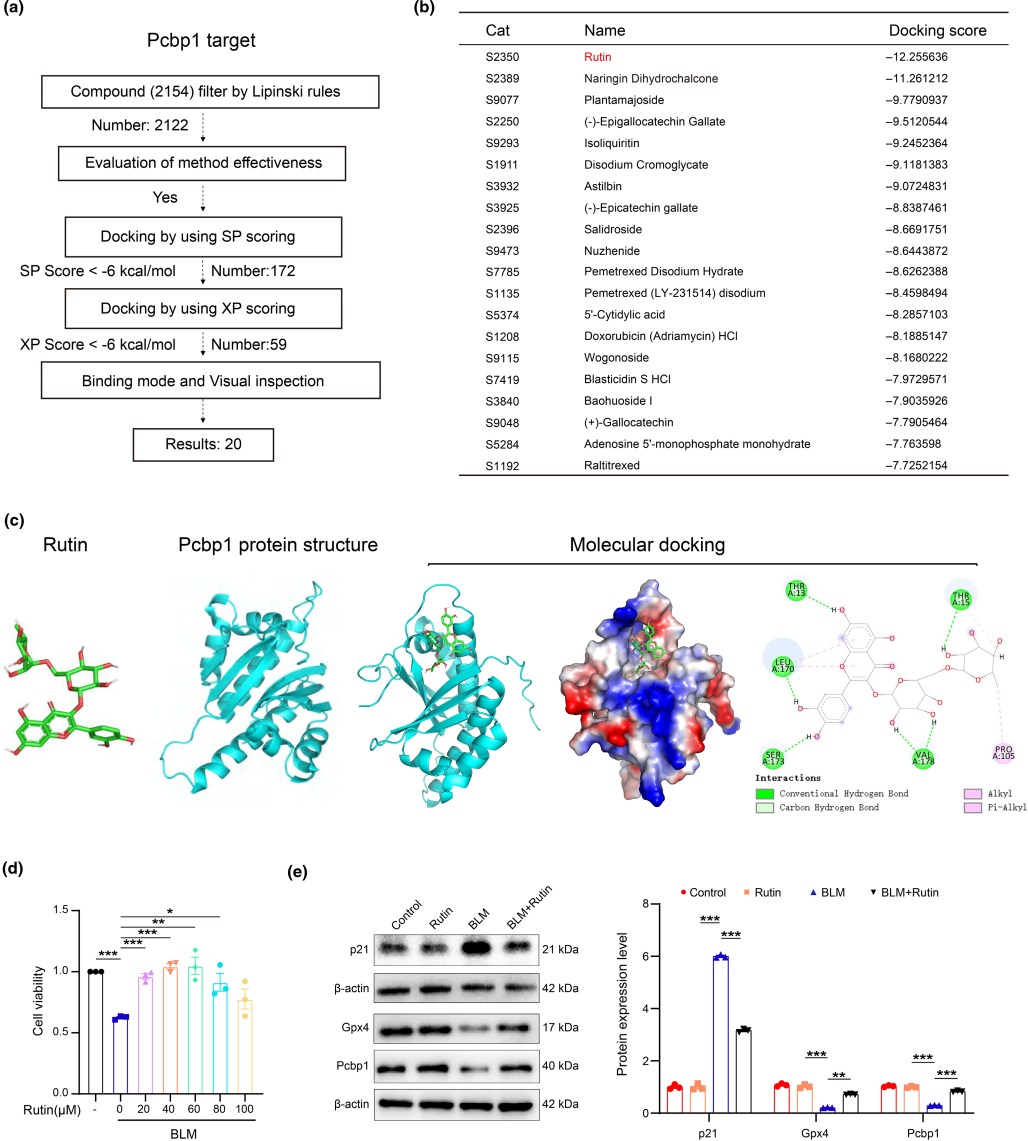
Rutin prevented macrophage senescence and ferroptosis by upregulating Pcbp1 in vitro. (a) Flow chart showing virtual screening‐based drug screening strategy using an Anti‐Aging Compound Library containing 2154 compounds. (b) The table shows the top 20 Anti‐Aging compounds targeting Pcbp1. (c) Rutin structure, Pcbp1 protein structure, and molecular docking of Rutin to the catalytic core of Pcbp1. (d) The CCK8 result shows the protection role of Rutin in drug‐induced senescent macrophage models in vitro. (e) Representative western blot and quantification analysis of p21, Gpx4, and Pcbp1 protein expression levels in the drug‐induced senescent macrophage model with or without Rutin in vitro. Data were represented as the mean ± SEM. *p < 0.05, **p < 0.01, and ***p < 0.001.

To identify potential therapeutics that could preserve Pcbp1 protein during ferroptosis, we employed a computer‐assisted drug screening strategy using an Anti‐Aging Compound Library containing 2154 compounds, as shown in the flow chart in Figure 7a. We used the Standard Molecular Property tool in the Maestro 11.9 platform to predict the physicochemical properties of these compounds. After filtering and screening, 2154 compounds were identified for further analysis. These compounds were evaluated based on binding energy fractions and key residues within the active site, yielding 20 compounds with strong binding energy (docking score <−7). Notably, among these, Rutin had a docking score of −12, indicating a strong binding affinity. This suggests that Rutin has a high potential to interact with Pcbp1 effectively (Figure 7b). The molecular docking of Rutin to the Pcbp1 protein is depicted in 3D structure (Figure 7c). Notably, Rutin binds within a specific pocket of the Pcbp1 protein, interacting through conventional hydrogen bonds, carbon hydrogen bonds, and hydrophobic interactions (alkyl and Pi‐alkyl interactions) with Threonine (THR), Leucine (LEU), Serine (SER), Valine (VAL), and Proline (PRO). To test whether this binding activates Pcbp1, we assessed the impact of Rutin on the viability of RAW264.7 macrophages treated with BLM. We found that concentrations between 40 and 60 μM offered significant protective effects, indicating that Rutin has the ability to preserve Pcbp1 function under senescence conditions (Figure 7d). Furthermore, Rutin treatment increased Pcbp1 protein levels, suggesting that it prevents Pcbp1 degradation. Consequently, Rutin rescued macrophage senescence, as evidenced by higher levels of Gpx4 protein and decreased p21 protein levels in macrophages treated with both BLM and Rutin (Figure 7e). Thus, Rutin might be a promising agent for modulating Pcbp1 function under aging conditions. Further, in vivo studies are necessary to confirm these effects and fully understand their therapeutic potential.

## 4 DISCUSSION

Our study elucidates the critical role of macrophage iron homeostasis and ferroptosis in the process of renal aging, enriching our understanding with comprehensive single‐cell RNA sequencing analyses. Our research not only highlights the importance of these processes in the aging kidney but also infers Stat1 as a key transcription factor and Pcbp1 as a crucial gene in regulating macrophage ferroptosis. This discovery provides a foundation for further detailed investigation into the functional roles of Stat1 and Pcbp1 in macrophage ferroptosis within the renal aging context. Additionally, our in vitro findings indicate a potential role for Rutin, a naturally occurring compound, in addressing age‐related renal fibrosis by modulating macrophage ferroptosis, thus presenting a novel therapeutic intervention that merits further investigation.

Iron dyshomeostasis is a hallmark of physiological aging, characterized by the gradual accumulation of iron (Slusarczyk et al., 2023). This accumulation leads to an increased intracellular generation of reactive oxygen species, culminating in the triggering of iron‐dependent cell death, known as ferroptosis, in aging organs (Li et al., 2023; Perdaens & van Pesch, 2021; Smith et al., 1997; Zeidan et al., 2021; Zheng et al., 2021). Iron overload in macrophages results in a proinflammatory state. These macrophages contribute to persistent chronic inflammation and induce paracrine senescence, ultimately impairing organ function (Sindrilaru et al., 2011; Zhou et al., 2018). Recent studies have suggested that the iron homeostasis of macrophages regulates systemic metabolic homeostasis and contributes to an overall healthier lifespan (Joffin et al., 2022). Our study systematically and deeply analyzed the functional changes of macrophages at each stage of renal aging at the single‐cell level, reinforcing the crucial role of macrophage iron metabolism and ferroptosis in aging. Additionally, our results showed a decrease in Pcbp1 expression in kidney macrophages of 12‐month‐old mice. We speculate that iron dyshomeostasis in macrophages occurs in the early stages of aging (middle‐aged or earlier).

However, further investigations are needed to substantiate our assumptions.

Our study has identified Pcbp1 as a key factor in both senescence and ferroptosis in macrophages, revealing that disturbances in Pcbp1 lead to immunosenescence. Initially recognized as an RNA‐binding protein, Pcbp1, also known as hnRNP E1, plays a crucial role in maintaining intracellular mRNA stability, participating in gene transcription, translation silencing, and enhancement (Philpott & Jadhav, 2019; Shi et al., 2008). Over time, Pcbp1 has also been found to act as an intracellular iron chaperone, actively maintaining the dynamic balance of the labile iron pool (Bayır et al., 2020). Hepatocyte‐specific deletion of Pcbp1 results in iron dyshomeostasis, mitochondrial damage, and ferroptotic cell death (Protchenko et al., 2021). Recent research has shown that decreased Pcbp1 expression in splenic and liver immune cells leads to iron dyshomeostasis and senescence, findings that are in line with our results (He et al., 2023). These findings suggest that Pcbp1 may serve as a universal anti‐immunosenescence gene across different organs.

Furthermore, our study inferred that Stat1, acting as an upstream transcription repressor, promotes ferroptosis in macrophages by repressing the transcription of Pcbp1, thereby contributing to chronic inflammation and organismal aging. Notably, the role of Stat1 in macrophage ferroptosis is not limited to its ability to suppress Pcbp1 expression. Stat1 typically functions as a transcription activator, leading to the transcription of multiple aging‐related genes. For example, Stat1 induces the transcription of genes like H2‐Ab1 (APP genes) and Wars (secretory marker genes), which mediate the aging phenotype in intestinal stem cells (Omrani et al., 2023). Therefore, our findings unveil a novel regulatory mechanism of iron homeostasis in macrophages via modulation of Pcbp1, offering potential insights for developing therapeutic strategies to combat aging‐related health problems.

Inspired by these results, the objective of our study shifted to the exploration of potential therapeutic agents to combat renal fibrosis in aging. In this endeavor, we discovered a promising interaction between Rutin and Pcbp1 through virtual screening and molecular docking, positioning Rutin as a potential anti‐aging agent. Known also as quercetin‐3‐O‐rutinoside or vitamin P, Rutin has been extensively reported for its role in kidney diseases, attributed to its anti‐oxidative and anti‐inflammatory effects (Dong et al., 2023; Hao et al., 2012; Negahdari et al., 2021). Crucially, several studies have highlighted Rutin's protective capabilities against aging‐related metabolic dysfunction and its role in extending the lifespan in mice, underscoring its potential as a drug candidate targeting aging processes (Li et al., 2016, 2022; Saafan et al., 2023). Our study contributes to this body of knowledge by proposing a novel anti‐aging mechanism for Rutin. It suggests that Rutin helps maintain iron homeostasis in macrophages and mitigates inflammation associated with ferroptosis in these cells by specifically regulating Pcpb1.

Our study presents certain limitations that warrant mention. Firstly, while we have explored the role of the transcription factor Stat1, the specific mechanisms by which it regulates Pcbp1 transcription remain unclear. Notably, Stat1 is predominantly recognized for its role in activating the transcription of immune response genes. However, it also possesses repressive functions, though less commonly. These repressive actions can manifest through various mechanisms, such as the direct binding of Stat1 to DNA and recruitment of histone deacetylases (HDACs), leading to chromatin modifications that suppress transcription (Nusinzon & Horvath, 2003). Secondly, the anti‐aging effects of Rutin have been established only through in vitro studies. Therefore, validating these effects in vivo is an essential step for future research. These limitations not only highlight areas for further investigation in our ongoing research but also open opportunities for other teams to contribute to this field.

In conclusion, our study provides a detailed analysis of the role of macrophages in renal aging, demonstrating their involvement in senescence and ferroptosis, and thereby contributing to aging‐related renal fibrosis. We inferred Stat1 as an upstream transcriptional repressor of Pcbp1, which is integral in initiating ferroptosis and inflammatory responses in macrophages. While our findings suggest that Rutin could potentially counteract immunosenescence and aging‐related renal fibrosis, it is important to note that these effects were observed in vitro. Therefore, the therapeutic efficacy of Rutin in these processes remains valid in vivo. This study lays the groundwork for future research to explore the potential of Rutin in clinical settings, emphasizing the importance of extensive in vivo research to thoroughly evaluate its therapeutic potential.

## AUTHOR CONTRIBUTIONS

Lingzhi Wu, Hui Peng, and Zhaoyong Hu designed the study and wrote the manuscript. Lingzhi Wu, Hongchun Lin, and Yuxiang Sun performed all the single‐cell data analysis. Lingzhi Wu and Yuebo Huang performed animal and cell culture experiments. Lingzhi Wu, Shuangshuang Shu, Ting Luo and Tiantian Liang performed the histological analysis. Lingzhi Wu, Weiyan Lai, Jialing Rao, and Shaomin Li participated in the interpretation of the results. Lingzhi Wu, Hui Peng, and Zhaoyong Hu finalized the manuscript. All authors critically read and commented on the final manuscript.

## FUNDING INFORMATION

This study was supported by the National Natural Science Foundation of China (81873613, 82170762 and 82370746), the Natural Science Foundation of Guangdong Province (2022A1515012637), the Fundamental Research Funds of Sun Yat‐sen University (82000‐18843406), and the Science and Technology Foundation of Guangdong Province of China (2021B1515230005), and the China Chronic Kidney Disease Management Innovation Program (202206080010).

## CONFLICT OF INTEREST STATEMENT

The authors declare no competing interests.

## Supporting information


Data S1

## Data Availability

The single‐cell transcriptomic data from the Cell Landscape database are available at http://bis.zju.edu.cn/cellatlas/ and PMID: 35929025. Other resources are available on reasonable request.
