# Supplementary material for: Macrophage iron dyshomeostasis promotes aging‐related renal fibrosis
Source: Aging Cell. 2024 Jul 17;23(11):e14275. doi: 10.1111/acel.14275 (PMC11561705; doi:10.1111/acel.14275)
Supplement: Supplementary file 1 — Data S1 [file ACEL-23-e14275-s001.zip › acel14275-sup-0001-SupinfoS1/Figure Legends.docx]

**Table S1.** Primer sequences in RT-qPCR.

**Table S2.** Macrophage age-related transcription factors during renal aging by SCENIC analysis.

**FIGURE S1.** GSVA score of the chemokine signaling pathway and cytosolic DNA sensing pathway in the immune system were calculated for all types of renal cells(a) and for macrophages across the age stage(b). DCTC: distal convoluted tubule cell; IC-CD: intercalated cell of collecting duct.

**FIGURE S2.** Heatmap for the upregulated overlapping DEGs (upper) and downregulated overlapping DEGs (lower) of macrophages. In red are

ferroptosis-related genes.

**FIGURE S3.** KEGG pathway enrichment of macrophages in 24 months in Tabula Muris Senis.

**FIGURE S4.** (a, b) Representative western blot and quantification of p21 and γH2AX protein expression in drug-induced senescent macrophage models in vitro. Data were represented as the mean ± SEM. **p < 0.01, and ***p < 0.001 **FIGURE S5.** Significant correlations between the Pcbp1, Hmox1, and Sat1 expression level and the GSVA score of leukocytes transendothelial migration pathway in Single-cell RNA sequencing data.

**FIGURE S6.** Gene expression levels of Stat1 from Single-cell RNA sequencing data.
