# Supplementary material for: Macrophage iron dyshomeostasis promotes aging‐related renal fibrosis
Source: Aging Cell. 2024 Jul 17;23(11):e14275. doi: 10.1111/acel.14275 (PMC11561705; doi:10.1111/acel.14275)
Supplement: Supplementary file 1 — Data S1 [file ACEL-23-e14275-s001.zip › acel14275-sup-0001-SupinfoS1/Supplementary file.pdf]

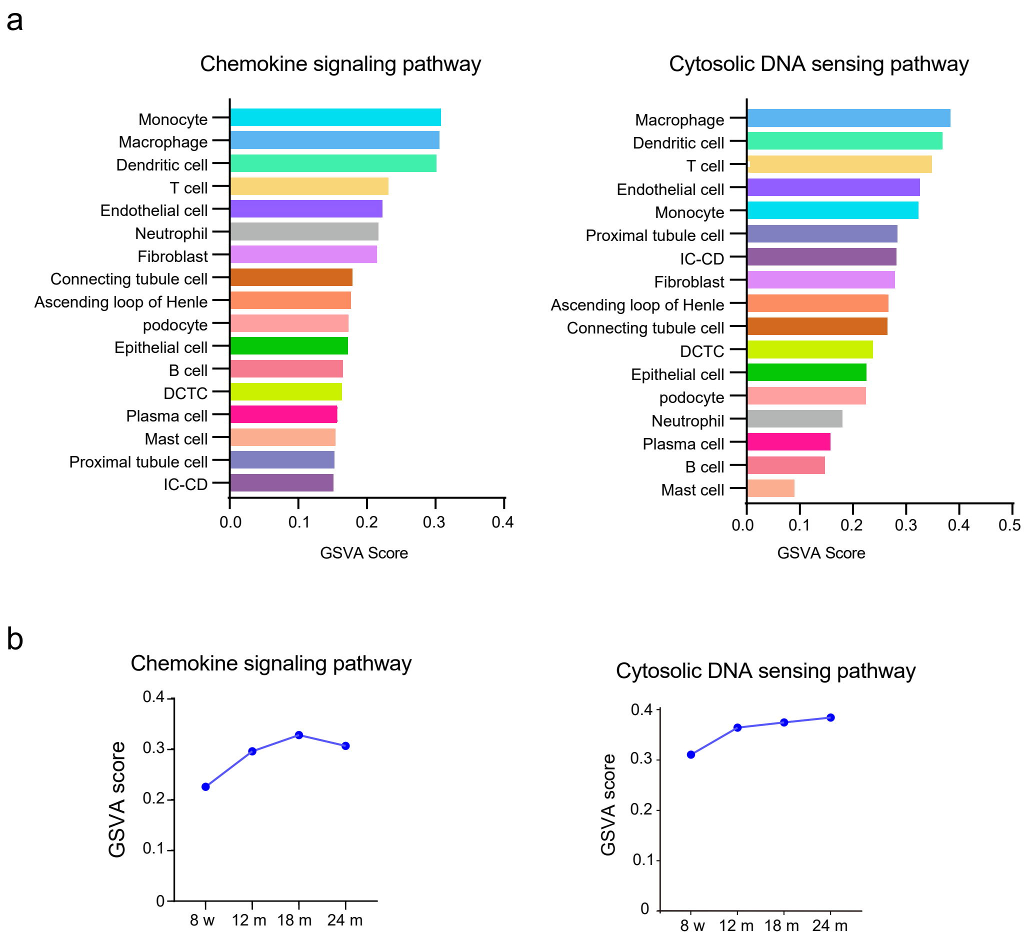

a
Chemokine signaling pathway
Monocyte
Macrophage
Dendritic cell
T cell
Endothelial cell
Neutrophil
Fibroblast
Connecting tubule cell
Ascending loop of Henle
podocyte
Epithelial cell
B cell
DCTC
Plasma cell
Mast cell
Proximal tubule cell
IC-CD
0.0 0.1 0.2 0.3 0.4
GSVA Score
Cytosolic DNA sensing pathway
Macrophage
Dendritic cell
T cell
Endothelial cell
Monocyte
Proximal tubule cell
IC-CD
Fibroblast
Ascending loop of Henle
Connecting tubule cell
DCTC
Epithelial cell
podocyte
Neutrophil
Plasma cell
B cell
Mast cell
0.0 0.1 0.2 0.3 0.4 0.5
GSVA Score
b
Chemokine signaling pathway
GSVA score
0 0.1 0.2 0.3 0.4
8 w 12 m 18 m 24 m
Cytosolic DNA sensing pathway
GSVA score
0 0.1 0.2 0.3 0.4
8 w 12 m 18 m 24 m

Up

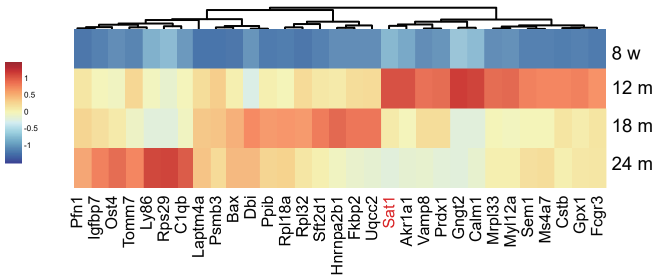
1
0.5
0
-0.5
-1
8 w
12 m
18 m
24 m
Pfn1
Igfbp7
Ost4
Tomm7
Ly86
Rps29
C1qb
Laptm4a
Psmb3
Bax
Dbi
Ppib
Rpl18a
Rpl32
Sft2d1
Hnrnpa2b1
Fkbp2
Uqcc2
Sat1
Akr1a1
Vamp8
Prdx1
Gngt2
Calm1
Mrpl33
Myl12a
Sem1
Ms4a7
Cstb
Gpx1
Fcgr3

Down

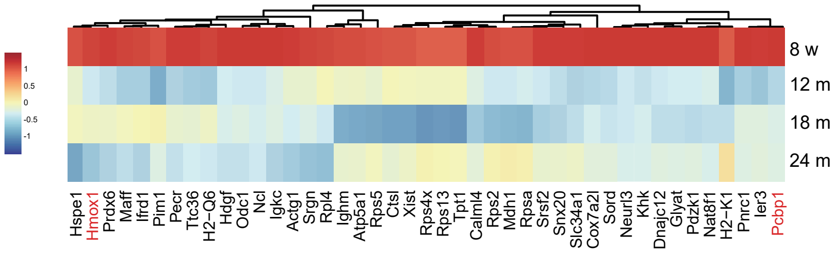
1
0.5
0
-0.5
-1
8 w
12 m
18 m
24 m
Hspe1
Hmox1
Prdx6
Maff
Ifrd1
Pim1
Pecr
Ttc36
H2−Q6
Hdgf
Odc1
Ncl
Igkc
Actg1
Srgn
Rpl4
Ighm
Atp5a1
Rps5
Ctsl
Xist
Rps4x
Rps13
Tpt1
Calml4
Rps2
Mdh1
Rpsa
Srsf2
Snx20
Slc34a1
Cox7a2l
Sord
Neurl3
Khk
Dnajc12
Glyat
Pdzk1
Nat8f1
H2−K1
Pnrc1
Ier3
Pcbp1

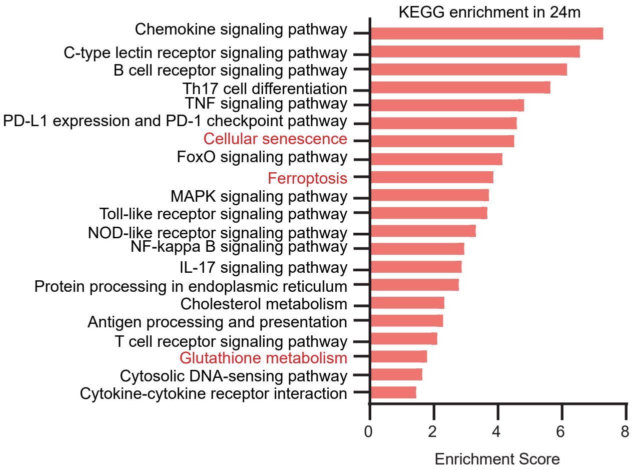

KEGG enrichment in 24m
Chemokine signaling pathway
C-type lectin receptor signaling pathway
B cell receptor signaling pathway
Th17 cell differentiation
TNF signaling pathway
PD-L1 expression and PD-1 checkpoint pathway
Cellular senescence
FoxO signaling pathway
Ferroptosis
MAPK signaling pathway
Toll-like receptor signaling pathway
NOD-like receptor signaling pathway
NF-kappa B signaling pathway
IL-17 signaling pathway
Protein processing in endoplasmic reticulum
Cholesterol metabolism
Antigen processing and presentation
T cell receptor signaling pathway
Glutathione metabolism
Cytosolic DNA-sensing pathway
Cytokine-cytokine receptor interaction
0
2
4
6
8
Enrichment Score

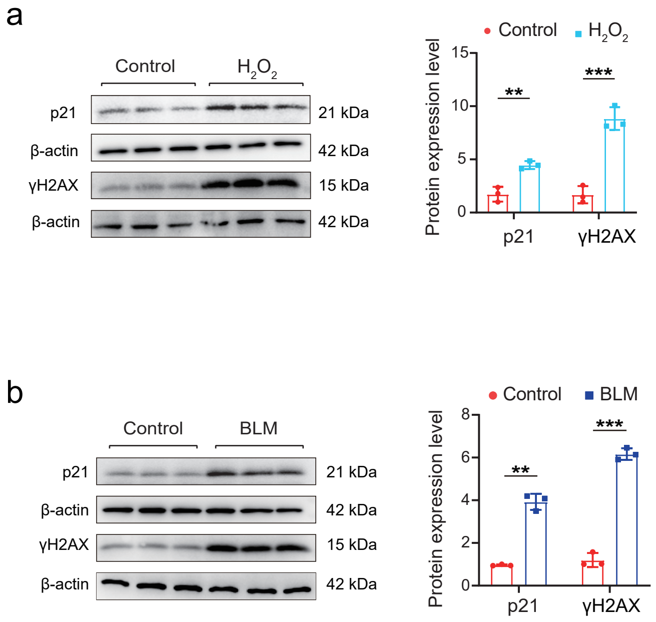

a
Control
H_2O_2
p21
21 kDa
β-actin
42 kDa
γH2AX
15 kDa
β-actin
42 kDa
Control
H_2O_2
Protein expression level
15
10
5
0
**
***
p21
γH2AX
b
Control
BLM
p21
21 kDa
β-actin
42 kDa
γH2AX
15 kDa
β-actin
42 kDa
Control
BLM
Protein expression level
8
6
4
2
0
**
***
p21
γH2AX

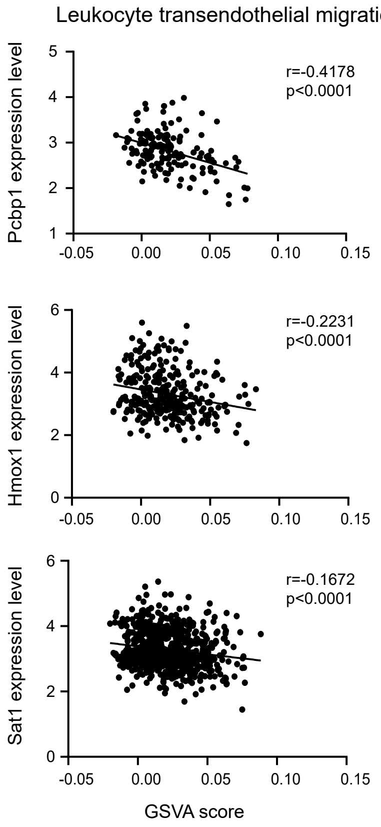
Leukocyte transendothelial migration
Pcbp1 expression level
r=-0.4178
p<0.0001
Hmox1 expression level
r=-0.2231
p<0.0001
Sat1 expression level
r=-0.1672
p<0.0001
-0.05
0.00
0.05
0.10
0.15
GSVA score

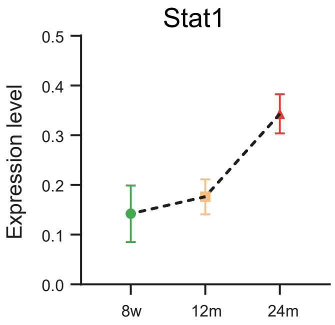

Stat1
Expression level
0.5
0.4
0.3
0.2
0.1
0.0
8w
12m
24m

Table S1. Primer sequences in RT-qPCR

IL-6 F	CCGGAGAGGAGACTTCACAG
IL-6 R	TCTGCAAGTGCATCATCGTT
TNF-α F	CAGGCGGTGCCTATGTCTC
TNF-α R	CGATCACCCCGAAGTTCAGTAG
IL-1 β F	GAAATGCCACCTTTTGACAGTG
IL-1 β R	TGGATGCTCTCATCAGGACAG
MCP-1 F	TAAAAACCTGGATCGGAACCAAA
MCP-1 R	GCATTAGCTTCAGATTTACGGGT
p21 F	CCTGGTGATGTCCGACCTG
p21 R	CCATGAGCGCATCGCAATC
Pcbp1 F	GGATTCGCCGGAATTGACT
Pcbp1 R	CATCCAAACTTGCCCAATAGC
Hmox1 F	AAGCCGAGAATGCTGAGTTCA
Hmox1 R	GCCGTGTAGATATGGTACAAGGA
Sat1 F	GGCTAAATTTAAGATCCGTCCA
Sat1 R	CATGTATTCATATTTAGCCAGTTCCTT
Stat1 F	TCACAGTGGTTCGAGCTTCAG
Stat1 R	GCAAACGAGACATCATAGGCA
ACTA2 F	GATCACCATCGGGAATGAACGC
ACTA2 R	CTTAGAAGCATTTGCGGTGGA
Vim F	CGTCCACACGCACCTACAG
Vim R	GGGGGATGAGGAATAGAGGCT
Snai1 F	CACACGCTGCCTTGTGTCT
Snai1 R	GGTCAGCAAAAGCACGGTT
β-actin F	ACTGGGACGACATGGAGAAG
β-actin R	GTCTCCGGAGTCCATCACAA

TFs	Target Gene
Bhlhe40-extended	1150
Ets2-extended	718
Mxd1-extended	176
Xbp1	348
Atf3	611
Elf4-extended	805
Bclaf1-extended	997
Fosb-extended	445
Crem-extended	376
Rel-extended	204
Atf3-extended	764
Etv3-extended	927
Xbp1-extended	542
Crem	177
Zbtb7a-extended	297
Bclaf1	656
Egr1-extended	744
Elf2-extended	798
Rel	137
Nfe2l2-extended	397
Klf6-extended	30
Elf4	510
Nr3c1-extended	642
Etv3	611
Maff-extended	110
Fosb	177
Nr3c1	308
Elk3	725
Fosl2-extended	212
Hdac2-extended	641
Stat3-extended	166
Gtf2f1-extended	1030
Elf2	536
Rela-extended	630
Gabpb1-extended	767
Elk3-extended	963
Cebpb-extended	666
Tgif1-extended	182
Sp3-extended	439
Bcl3-extended	83
Elf1	471
Jund-extended	354
Etv5-extended	830
Ets2	54
Jun-extended	101
Ets1-extended	917
Jund	18
Etv6-extended	863
Zmiz1-extended	893
Nfkb2-extended	48

Tgif1	32
Egr1	48
Irf1-extended	771
Batf-extended	147
Jun	30
Fli1-extended	660
Ctcf-extended	653
Sap30-extended	377
Junb-extended	295
Junb	42
Yy1-extended	911
Spic-extended	858
Spic	266
Nr1h3-extended	46
Nr1h3	19
Gabpa-extended	826
Bmyc-extended	667
Stat1-extended	795
Taf1	856
Taf1-extended	1121
Srebf2-extended	806
Fos	50
Fos-extended	188
